# Glioblastoma immunotherapy in the context of the aging immune system: a systematic review and meta-analysis

**DOI:** 10.1007/s11060-025-05395-1

**Published:** 2026-01-12

**Authors:** Jack M. Shireman, Simon Ammanuel, Lingxin Cheng, Emily Distler, Yilong Tao, Christina Kendziorski, Mahua Dey

**Affiliations:** 1 Department of Neurosurgery, University of Wisconsin School of Medicine & Public Health, UW Carbone Cancer Center, 600 Highland Ave, Madison, WI 53792 USA; 2 Department of Biostatistics and Medical Informatics, University of Wisconsin School of Medicine and Public Health, Madison, WI USA

**Keywords:** Aging, Immunotherapy, Glioblastoma, Clinical trials

## Abstract

**Purpose:**

Immunotherapy has yet to meaningfully translate to more complex solid tumors, such as Glioblastoma (GBM), which is a disease of old age with a median diagnosis age of 64. Despite this clear age bias, very little research has been conducted on the interplay between the aging immune system and its impact on the efficacy of immunotherapy.

**Methods:**

A literature search and meta-analysis was performed to quantify the role of the aged immune system during immunotherapy treatments in GBM. Registered clinical trials conducted from Jan 2000-April 2025 were analyzed and risk ratio of death at 1 year post treatment was calculated using patient level data for participants aged 65 and older and 64 and under.

**Results:**

Across 30 total studies and 556 patients’ data revealed a significantly higher risk of death (RR: 1.29: (1.09-1.53), *p* = 0.0040) at or before 1 year post immunotherapy treatment in the aged population compared to the young population. This risk was even larger in newly diagnosed GBM (RR: 2.24: (1.39-3.61), *p* = 0.0026). Finally, when examining the ages of patients enrolled in GBM immunotherapy clinical trials we found a significant bias towards enrolling younger patients. This bias was not present among lung cancer, also a disease of older adults, immunotherapy clinical trials.

**Conclusion:**

These data highlight that the aging of the immune system may play a role in the response to immunotherapy and trial designs with better tracking and reporting of this variable will allow for a more careful examination of this effect and overall successful immunotherapy development.

**Supplementary Information:**

The online version contains supplementary material available at 10.1007/s11060-025-05395-1.

## Introduction

Immunotherapy has been a revolution in cancer care with the ability to deliver long term therapeutic efficacy once thought impossible. Unfortunately, despite its success, immunotherapy has faced setbacks when applied to more cellularly dense and complex solid tumors slowing its progress as a pan-cancer therapy [1–3]. Many aspects of solid tumors present challenges that a successful immunotherapy must overcome, chief among those, is that they are largely diagnosed in older individuals with an aged and weakened immune system.

The human immune system begins to decline around the age of 50 with significant thymic atrophy taking over by the age of 65 [4–7]. At this point there are major shifts in the overall make up and balance of circulating T-cells with significant increase in senescent T-cells and losses in circulating effector memory cells thought to underlie the weakened vaccine response seen in the elderly [7–11]. Recognizing these aging related changes at the population level, the CDC recommends vaccine boosters for individuals over the age of 65 [12–14].

A primary promise of cancer immunotherapy is harnessing the intrinsic power of the immune system to identify and eliminate only the cancerous cells while sparing other normal cells, something conventional therapies such as radiation and chemotherapy lack. A critical disconnect occurs in clinical decision making when a therapy designed to leverage the immune system is given to an aged individual with an already weakened immune system at baseline, let alone the immunosuppression caused by an advanced solid tumor. Numerous solid tumors, including one of the most immunosuppressive tumors, glioblastoma (GBM), have had several modalities of immunotherapy fail to demonstrate efficacy in large randomized controlled trials [15–18] after showing somewhat positive results in early phase trials. This lack of translation of early phase clinical trial results to larger phase III trials, and therefore to the disease population level, results in tens of millions of dollars invested without any therapeutic gain [19–22]. To understand how immunotherapy will function in a cancer that has an average age at diagnosis of 64 years, we need to understand how immunotherapy performs in the aged immune system.

To examine this question, we conducted a systematic literature search and meta-analysis aimed at analyzing the overall survival in aged individuals compared to young individuals when treated with immunotherapy to target GBM. We found a possible increased risk of death at or before 1 year in aged individuals across all immunotherapies in both newly diagnosed and recurrent GBM, with the largest effect in newly diagnosed GBM. Our data also suggests that GBM clinical trials at the phase II and III levels are heavily skewed towards enrolling younger and healthier patients, a bias that is not seen in comparable lung cancer clinical trials. These results underscore the need for more nuanced investigation of the efficacy of immunotherapy in aged patients and a more judicious recruitment of representative patients in early phase clinical trials.

## Methods

### Clinical trial eligibility criteria and search strategy

A literature search was undertaken by the authors and replicated in triplicate according to the Preferred Reporting Items for Systematic Reviews and Meta-Analyses (PRISMA) guidelines (checklist attached as supplementary material). The search was formatted according to the Population, Intervention, Comparison, Outcome, Study Type (PICOS) question method: In glioblastoma patients, is there a difference in immunotherapy efficacy in aged (65 or older) vs. younger cohorts (64 or younger)? The outcome sought/examined was overall survival (OS) at 1 year post therapy. The criteria for this search are outlined in supplementary Table 1. The initial literature search was completed using PubMed and the search was confirmed using the Cochrane database. The search terms included were “glioblastoma and immunotherapy”, “glioblastoma and virus”, “glioblastoma and cellular vaccination”, “glioblastoma and peptide vaccination”, “glioblastoma and checkpoint inhibitors”, “glioblastoma and PD-1”, “glioblastoma and CTLA-4”, “glioblastoma and cytokine therapy”, “glioblastoma and CAR-T”, “glioblastoma and immune stimulation”, “glioblastoma and immune microenvironment” and “glioblastoma and dendritic cell vaccination”. In PubMed the applied filters were phase II, III, IV with a date range from January 1st, 2000, to April 1st, 2025. The positive search results were verified between authors JMS, SA, ED and YT. JMS collected the data form the manuscripts and SA verified the collection as accurate. Data was retrieved from the study’s main figures and tables or supplemental information available with article publication. A flowchart demonstrating the search results is included in supplementary Fig. 1. No other automated search tools were utilized. No missing data or summary statistics were present in our selected trials, and data from trials was visualized in grouped fashion using statistical software package meta to generate forest plots. This review was not pre-registered, and a pre-protocol was not prepared.

### Data collection

All data were extracted from the main manuscript, figures, and supplemental material within the published trials by study authors, no automated or manual extraction tools were utilized. Risk of death at or before 1 year was calculated in R or heterogeneity score (I^2^), and 95% confidence intervals (methods below). Data extraction was done by three independent investigators (JMS, SA, and ED) and extracted data was confirmed by final review (JMS/MD). The primary outcome collected was overall survival (OS) at 1 year post therapy in both primary and recurrent GBM. Phase III clinical trials for analysis were selected based on similar inclusion and exclusion criteria as Phase II clinical trials. All phase III trials included had published age analysis and HR calculation.

### Statistical analysis

Statistical analyses were conducted using R Version 4.4.2. The meta-analyses, tests for heterogeneity, and publication bias analysis was performed using the R package meta version 8.2–1 [23]. OS at 1 year was collected and stratified according to the age bins specified above. In total, 30 studies met the inclusion criteria (sup Fig. 1). These studies included 556 patients diagnosed with either newly diagnosed GBM or recurrent GBM and treated with immunotherapy on a clinical trial protocol from Jan 2000 to April 2025 (Table 1). Using both a random and common effects model risk of death at or before 1 year post immunotherapy was calculated as a pooled risk ratio across all 30 studies. The random effects model calculated by the Mantel-Haenszel (MH) method is reported in the results section. To quantify the effects of age, individual trial participants were grouped into the control condition or experimental condition based on age with 65 and over being considered experimental (98 total patients) and 64 or younger being considered control (458 total patients). Heterogeneity between our studies was also assessed with the I^2^ statistic that is derived from the chi-squared distribution. An I^2^ statistic > 50% indicated the presence of heterogeneity. Publication bias was also assessed in our analyses using inspection of contoured funnel plots. For comparison of data across GBM and Lung cancer clinical trials a 2-tailed Welch’s T-test was utilized assuming unequal standard deviations between populations.

For calculation and fit of the linear model analysis for individual patient database R was utilized with patients’ original data supplied by the publications and included if data was complete with age, gender, IDH-mutation status, and MGMT methylation status, along with OS. All predictors were tested together and in a standard linear model with prediction of OS as the main outcome. ANOVA was conducted in base R and reported in the results.

## Quality evaluation of clinical trials

Trials used for analysis in this study were methodologically evaluated according to the revised Cochrane risk-of-bias tool for randomized trials. This system categorizes the studies as low, unclear, or high risk of bias, according to defined parameters [24]. All trials in the study score within the same high risk of bias category because of their similarity in design (sup Fig. 2). This increased risk of bias is mostly due to the lack of true randomization in all the trials captured for this analysis. Although there are larger randomized trials testing a number of these therapies, they rarely include individualized data or age breakdowns of their participants.

## Results

### Risk of death at or before 1 year post-immunotherapy treatment in GBM is significantly greater in aged individuals

**Fig. 1:**
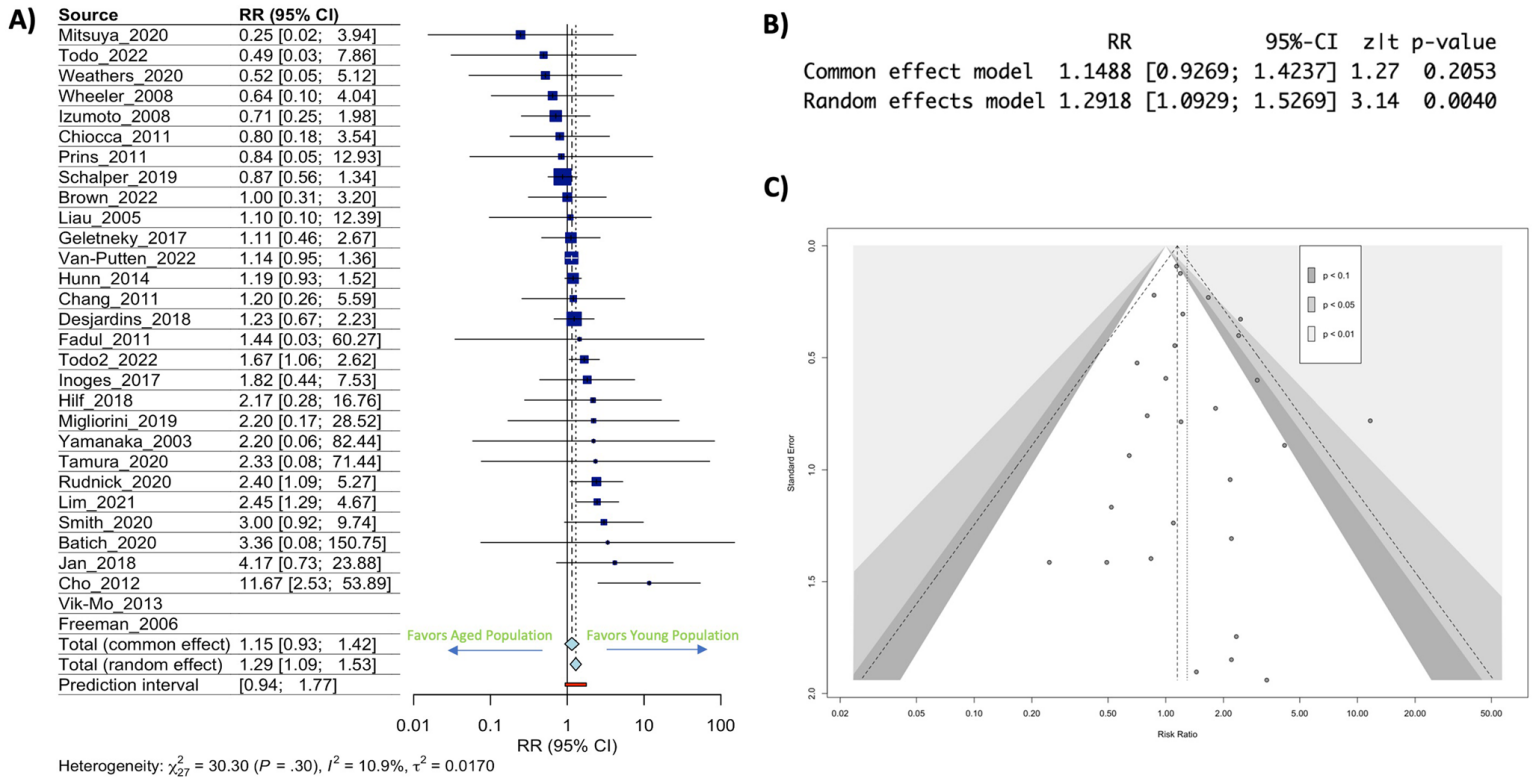
Risk of death at or before 1-year post-immunotherapy treatment in GBM is significantly greater in aged individuals. **(A)** Forest plot depicting risk ratio of death at or before 1 year in aged individuals with confidence intervals for each included clinical trial. **(B)** Statistics from the common and random effect model calculations **(C)** Funnel plot for all included clinical trials

In examining aged patients’ risk of death at or before 1 year, across both newly diagnosed and recurrent GBM, the calculated risk ratio of 1.29 (95% CI: 1.09–1.53) was significantly higher (*p* = 0.0040) in the aged group compared to the younger group representing an increased risk of death at or before 1 year in aged individuals undergoing immunotherapy treatment for GBM (Fig. 1A & B). Publication bias risk analysis demonstrated little effect of publication heterogeneity/bias in our analysis (I^2^ statistic (10.9%) (Fig. 1C). To address the analytical limitation of this type of meta-analysis, we performed single patient level analysis of the known predictors (MGMT methylation status, IDH mutation status, and gender) of OS across these trials. We identified all individuals with newly diagnosed GBM (the largest RR increase) who had these data points available in our included studies. Of the 213 total patients in the included studies, only 96 patients had data reported on all predictive factors and were included in the analysis. ANOVA demonstrated no predictive value of any of the included variables for OS (sup Fig. 3A & B). We also examined published phase III clinical trials that had calculated HRs for age within their study designs and identified only 4 trials that had conducted this type of analysis, and the data demonstrated no significant effect of age on immunotherapy outcomes (sup Fig. 4A, B &C). However, all these trials included disproportionally fewer patients over age 65 hampering any meaningful conclusions from this statistical analysis. These results indicate that age may play a role in survival post immunotherapy across a broad slate of immunotherapy treatments in GBM patients, but that more data and better reporting is needed to fully understand this complex effect.

### Immunotherapy efficacy in newly diagnosed GBM is more significantly impacted by increased age than recurrent GBM

**Fig. 2:**
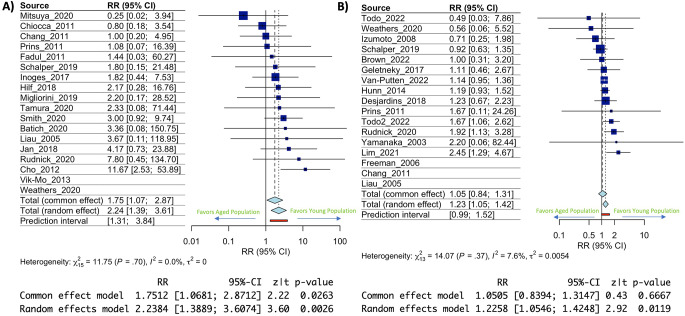
Immunotherapy efficacy in newly diagnosed GBM is more significantly impacted by increased age than recurrent GBM. **(A)** Forest plot depicting risk ratio of death at or before 1 year in aged individuals with newly diagnosed GBM. Confidence intervals for each included clinical trial and statistical output of the model calculations are below. **(B)** Forest plot depicting risk ratio of death at or before 1 year in aged individuals with recurrent GBM. Confidence intervals for each included clinical trial and statistical output of the model calculations are below

A large base of literature has hypothesized that clinical trial intervention with novel immunotherapies would be more beneficial if given when GBM is newly diagnosed as opposed to treatment solely at recurrence [25–27]. To examine the role age plays in this effect our participant data was separated according to diagnosis stage with trials including only newly diagnosed GBM or trials including only recurrent GBM. Results showed a more significant effect of age on risk of death at or before 1 year post immunotherapy treatment in newly diagnosed GBM (RR: 2.34, 95% CI: 1.39–3.61, *p* = 0.0026) (Fig. 2A) compared to recurrent GBM (RR:1.23, 95% CI: 1.05–1.42, p 0 0.0119) (Fig. 2B). In both cases the I^2^ statistic was less than 10% indicating very little publication bias/heterogeneity (Fig. 2A & B). This data highlights an over 2x risk of death at or before 1 year for newly diagnosed GBM aged participants (> 65) being treated with an immunotherapy. Comparison of risk ratio’s plus 95% confidence intervals (CI’s) also demonstrated little overlap (sup Fig. 5). Although there was still a significant age effect in recurrent GBM patients, the largest effect seen within the subgroup analysis was on newly diagnosed GBM suggesting an increased role of age at that disease timepoint.

### Vaccine based immunotherapies may be more impacted by age than non-vaccine-based immunotherapies

**Fig. 3:**
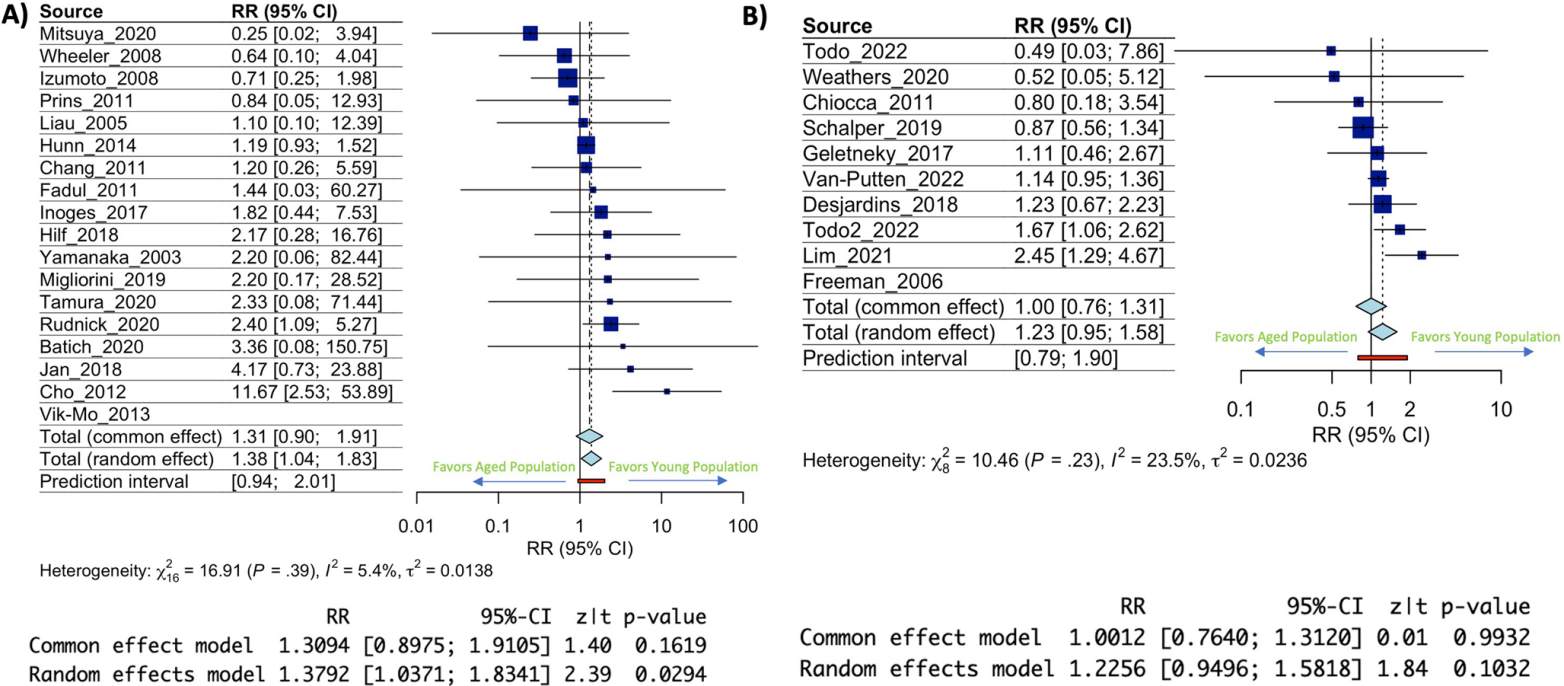
Vaccine based immunotherapies may be more impacted by increased age than non-vaccine-based immunotherapies. **(A)** Forest plot depicting risk ratio of death at or before 1 year in aged individuals treated with vaccine-based immunotherapy. Confidence intervals for each included clinical trial and statistical output of the model calculations are below. **(B)** Forest plot depicting risk ratio of death at or before 1 year in aged individuals treated with non-vaccine-based immunotherapy. Confidence intervals for each included clinical trial and statistical output of the model calculations are below

As the human immune system ages different points of failure occur. One well studied point of failure is the response to vaccinations and the generation of vaccine-based immunity in aged individuals [12–14]. This effect has been well documented in vaccinations having to do with foreign pathogens such as Flu, COVID, or Shingles [28–34]; however, it is underexplored in terms of response to vaccines for cancer. To study this, we categorized our included immunotherapy trials into either a vaccine-based immunotherapy, which included therapies such as dendric cell vaccines (DC vaccines) and peptide vaccines, or non-vaccine-based immunotherapy which included all other immunotherapies from our included trials list. We observed a statistically significant increase in risk ratio of 1.38 (95% CI: 1.04–1.83, *p* = 0.0294) (Fig. 3A) between aged and young patients in cohorts of vaccine-based immunotherapy. Conversely, we observed a statistically insignificant increase in risk ratio of 1.23 (95% CI: 0.95–1.58, *p* = 0.1032) between aged and young patients in cohorts of non-vaccine-based immunotherapy (Fig. 3B). When risk ratios and their 95% CIs were compared, we saw significant overlap between the groups indicating the effect may be weak or nonexistent given the current data (sup Fig. 6). The smaller sample size in the non-vaccine-based immunotherapy cohort did result in an increase in the I^2^ statistic, however, it remained under 30% indicating low to no publication bias/heterogeneity effect. These data suggest a possible driving factor behind the effects seen in this analysis is the effect of age specifically on vaccine-based immunotherapy as opposed to other therapy modalities, though further and more targeted studies will be needed to explore this possibility.

### Age based recruitment bias is present in the GBM clinical trial populations but not present in lung cancer clinical trial populations

**Fig. 4:**
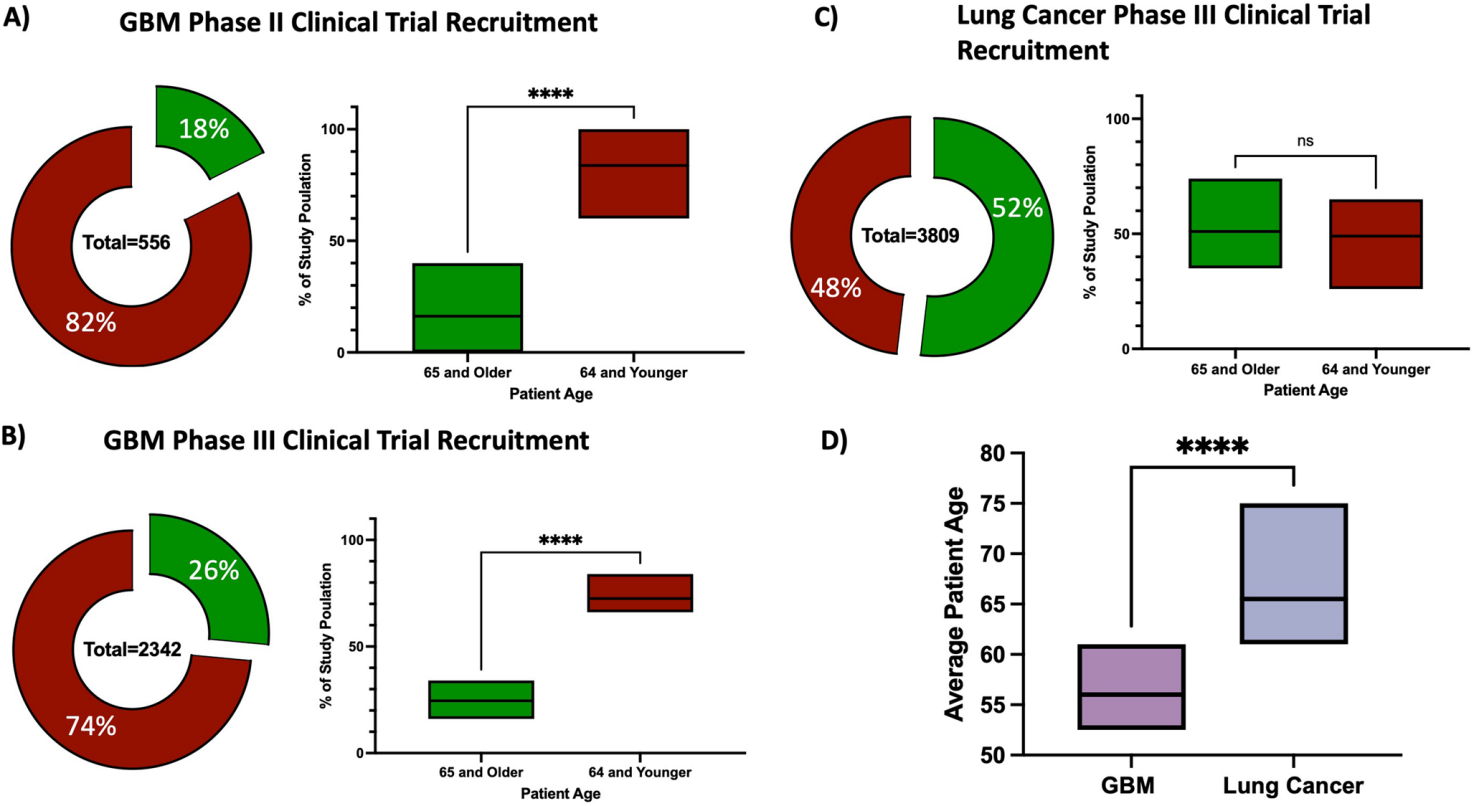
Age based recruitment bias is present in the GBM clinical trial populations but not present in lung cancer clinical trial populations. **(A)** Pie chart and box plot depicting the age of participants enrolled in phase II immunotherapy clinical trials in GBM **(B)** Pie chart and box plot depicting the age of participants enrolled in phase III immunotherapy clinical trials in GBM **(C)** Pie chart and box plot depicting the age of participants enrolled in phase III immunotherapy clinical trials in lung cancer. **(D)** A boxplot representing the average age of a patient included in a phase III GBM immunotherapy trial compared to a similar trial in lung cancer. **** *p* < 0.0001

When conducting the literature review and assembling this dataset it became obvious that GBM clinical trials, especially at the phase II levels, recruit heavily from younger patient cohorts (below 65 years of age) despite the average age of diagnosis for GBM being 64. In our patient population across 556 patients only 18% were age 65 or older (Fig. 4A). When represented as proportions of each individual study population there was a highly significant difference between the proportion of aged individuals and young individuals across GBM clinical trials (17% vs. 82% respectively, *p* < 0.0001) (Fig. 4A). In fact, in the 30 studies included for analysis in this meta-analysis no study included more than 40% aged individuals in its overall population. Phase II clinical trials have smaller recruitment targets than larger phase III trials. To examine if these larger trials also suffered from an age recruitment bias data 9 immunotherapy phase III clinical trials in GBM were examined (Table 22). These results show similar age recruitment bias with only 26% of enrolled participants over the age of 65 across 2342 patients (Fig. 4B) as well as proportionally lower aged patients compared to young patients (26% vs. 73% respectively, *p* < 0.0001) (Fig. 4B). To understand if this was a pervasive bias in clinical trials with diseases diagnosed later in life, we examined lung cancer clinical trials conducted using similar literature search criteria as was used to screen for GBM studies (supp Table 2). According to SEER data* the median age of diagnosis for lung cancer is roughly 70 years old and their clinical trial population represents that with a nearly 50/50 split on recruitment of patients above and below 65 (Fig. 4C). Furthermore, we applied the same analysis examining the proportion of aged individuals across each study and found no statistically significant difference (Fig. 4C) with more than 50% of lung cancer patients in phase III immunotherapy clinical trials being over the age of 65. Finally, when comparing the mean age of participants enrolled in similar phase III immunotherapy clinical trials in GBM or lung cancer there was a significant difference in average participant age with GBM having a mean age of 57 years while lung cancer had an age of 67 (*p* < 0.0001) (Fig. 4D) This underscores the need for proper age balancing in GBM clinical trials, especially ones utilizing immunotherapy.

## Discussion

Immunotherapy represents a significant and effective tool in the arsenal of physicians but there are still many nuances in its use that remain understudied. Although the impact of aging on the immune system is well known there is little clinical research reporting on this impact when it comes to immunotherapy as a treatment for cancers. The biology behind an aging immune system is highly relevant as the general US population is rapidly aging and cancer, including GBM, is a disease of older adults [35–37]. Although aging has a profound effect on immune system this biological effect is not clearly captured or robustly studied in the clinical setting. Our data analysis demonstrates that in GBM age likely plays a role in a response to immunotherapies in both newly diagnosed and recurrent tumors, however, the data reported from current clinical trials is less than ideal for truly understanding this complex variable and its role in the success of immunotherapy.

Data from this study also uncovers a large bias in the recruiting of GBM clinical trial participants with studies tending to recruit younger patients rather than older ones. This bias at earlier phase II trial levels only serves to reinforce spending more research funding on translating the therapies to larger phase 3 trials which are anywhere from 2-10x the cost, depending on the estimate [19–22]. If these phase III trials are only being run after “successful” phase II trials on a patient population not representative of the average GBM patient, then the therapy is bound to fail at the phase III level and subsequently in the general population. These failures in translation from phase II to III are well documented in the GBM immunotherapy space. Although we did find more age balance in the examination of phase III GBM clinical trials when compared to phase II trials, there was still a significant age bias present. Importantly, this age bias was not present in lung cancer immunotherapy clinical trials at the phase III level, indicating that it is possible to recruit and study an aged population in the clinical trial setting, although it is important to knowledge that lung cancer trials do recruit from a significantly larger portion of diagnosed patients than a typical GBM trial would.

Another pressing question is whether this age effect may be driven by a specific type of immunotherapy rather than all immunotherapies in general. Of the 30 trials included in the analysis a significant portion of them were cell-based vaccines or peptide vaccines. When vaccine based clinical trials were separated out from the rest of our trial population there was a significant increase in risk ratio in the vaccine-based trials compared to the non-vaccine-based trials, however, the comparison between the risk ratios of aged and young participants showed significant overlap likely because of the disproportionate size of the aged vs. young participant groups within these trials. Longstanding basic science and clinical research has implicated age as a main driver for reduced vaccine response, with the CDC recommending more frequent vaccine doses for many elderly individuals across a wide variety of diseases. Scientifically these results make sense with a vaccine-based response typically engaging more facets of the immune system than say an antibody-based therapy. Vaccine based immunity requires not only a well-presented antigen but also a clonal expansion of effector cells, generation of memory cells, and efficient trafficking of these cells to the area of the antigen all of which are known to be compromised with age [30, 31, 33, 34]. It’s possible effects like this need to be considered more when recommending certain types of immunotherapies to older populations. In fact, one lesson that can be learned from the GBM clinical trial landscape in general is that one size fits all treatments seem to be largely ineffective and consistently produce failed clinical trials. Here immunotherapy itself has the advantage of a more personalized therapeutic option, however, we cannot simply automatically consider everyone’s immune system ready and equal to respond.

Although age impacts all forms of therapy, immunotherapy may be uniquely susceptible due to the well-known deterioration of the immune system with age as opposed to other forms of therapy such as chemotherapy or radiation. Age itself needs to be more incorporated into GBM clinical trial design and our analysis from lung cancer immunotherapy clinical trials shows that this is possible. Furthermore, additional data reporting from larger multicentered trials on the age ranges of the trial cohorts and including age as a cofactor analysis by default will help advance this understudied phenomenon. Ultimately biasing clinical trial enrollment towards younger and healthier patients may produce transiently better trial outcomes, however, it significantly limits it generalizability to a “typical” GBM patient who is unlikely to fair as well. Ideally with large more coordinated multicenter trials it will be possible for a disease with lower prevalence like GBM to recruit more representative and larger patient cohorts for new clinical trials.

Although our work is robust based on the current landscape of the available datasets; there are several limitations of this study. Firstly, age is a complex topic to study because it is associated with several other comorbidities, some having to do with the immune system and some unrelated. It has been well documented that age is an independent negative prognostic marker for GBM [36, 38–41]. Secondly, all the clinical trials we were able to include in the analysis are at a very high risk of bias due to their lack of randomization and blinding which should temper the resulting conclusions. All available phase II trials do not include a control arm and thus do not calculate HR’s making a traditional meta-analysis difficult. When conducting an individualized analysis or examining phase III trials with age calculated HRs the number of patients and/or quality of data included degrades significantly. Our results highlight the possibility of a significant age effect on outcomes in immunotherapy and demonstrate a critical need for better reporting and tracking of this effect to improve the trial landscape and overall success. In fact, we believe it’s worth nothing that even in the biased recruitment landscape of the currently conducted phase II and III trials the often-healthier aged individuals still respond worse at 1 year post treatment than younger individuals and this is likely to be more pronounced if an average aged GBM patient population was included. On the other hand, novel immunotherapy modalities specifically addressing an aging immune phenomenon such as immune cell senescence or thymic atrophy [42, 43] may be more successful in a trial that includes appropriately aged individuals. Importantly, we will never be able to study these effects appropriately if we are not including and tracking age as an independent biological variable for these clinical trials.

Overall, the findings from this study show that age may have an impact on survival during immunotherapy treatment for GBM. They highlight a bias in recruiting younger and healthier patients for clinical trials. More nuanced study and examination of these possible effects are critical in advancing the science behind immunotherapy and making these treatments more generalizable to the general population of individuals diagnosed with GBM.

**Table 1:** Phase II GBM immunotherapy trials included in this meta-analysis

Author (first)	Year	Immunotherapy type	Primary or recurrent	% of aged patients in trial	Total *N*
Inoges	2017	DC Vax whole lysate	primary	35	31
Weathers	2020	CMV specific expanded T-cells	mix	5	20
Cho	2012	DC Vax whole lysate	primary	6	18
Batich	2020	CMV targeted DC vax	primary	22	23
Lim	2021	ex-vivo expanded and activated t and NK cells	recurrent	7	14
Izumoto	2008	Peptide Vaccine for WT1 antigen	recurrent	19	21
Geletneky	2017	Oncolytic Parvovirus	recurrent	28	18
Wheeler	2008	DC Vax whole lysate	mixed	15	34
Tamura	2020	Peptide vaccine to VEGF	primary	25	4
Fadul	2011	DC Vax whole lysate	primary	40	10
Migliorini	2019	multi peptide vaccine	primary	31	16
Yamanaka	2003	DC Vax whole lysate	recurrent	29	7
Chang	2011	Dc Vax autologous	mix	14	14
Todo	2022	Oncolytic herpes virus	recurrent	21	19
Todo	2022	Oncolytic herpes virus	recurrent	8	13
Schalper	2019	Neoadjuvant Nivolumab	mixed	17	30
Vik-MO	2013	DC Vax targeted to stem cells	primary	0	7
Jan	2018	DC Vax autologous	primary	7	27
Hunn	2014	Dc Vax autologous	recurrent	14	14
Rudnick	2020	DC Vax + glidel wafer	mixed	22	23
Desjardins	2018	poliovirus	recurrent	13	61
Hilf	2018	neoantigen peptide vaccine	primary	19	16
Chiocca	2011	Oncolytic herpes virus	primary	20	10
Prins	2011	DC Vax	mixed	13	23
van Putten	2022	convection enhanced delivery of Oncolytic virus	recurrent	16	19
Freeman	2006	Oncolytic virus	recrurent	0	11
Liau	2005	DC vaccine	mixed	8	12
Brown	2022	CART with steroid resistance	recurrent	17	6
Smith	2020	CMV specific expanded T-cells	primary	14	21
Mitsuya	2020	DC Vax synthetic cocktail pulse	primary	36	14

**Table 2:** Phase III GBM immunotherapy trials examined for age bias

Author (first)	Year	% of aged patients in trial	Mean/median age	Total *N*	Incudes age subgroup analysis?
Reardon	2020	23	55	184	no
Reardon	2020	16	55	185	
Lassman	2025	N/A	59.5	80	no
Lassman	2025	N/A	61	79	
Kong	2016	N/A	53	91	no
Kong	2016	N/A	53	89	
Narita	2018	N/A	52.5	58	Yes, found that age < 70 was a HR ratio increase but < 50 or 50–69 wasn’t significantly different
Narita	2018	N/A	59	30	
Liau	2023	22	N/A	232	Yes, found that both ages > and < 65 favored DC vax pop
Liau	2023	N/A	56	64	
Westphal	2015	N/A	53	71	No
Westphal	2015	N/A	56	71	
Lim	2022	32	60	358	Yes, and found that age does worse in the immunotherapy group but only over 75, 65–75 wasn’t significant
Lim	2022	34	60	358	
Weller	2017	23	59	371	Yes, above and below 65 no difference in either SRD or MRD population.
Weller	2017	23	58	374	
Omuro	2022	32	60	280	No
Omuro	2022	26	56	280	

## Supplementary Information

Below is the link to the electronic supplementary material.

Supplementary Material 1: Inclusion/exclusion criteria for the meta-analysis and search engine terms.

Supplementary Material 2: Phase III lung cancer immnunotherapy trials examined for age bias

Supplementary Material 3: PRISMA flow chart.

Supplementary Material 4: Cochrane Risk of Bias Analysis.

Supplementary Material 5: Linear regression analysis of predictors for OS in GBM immunotherapy trials. A) Regression Analysis B) ANOVA

Supplementary Material 6: HR comparison of Phase III clinical trials including an age bin analysis. A) Young Patients (65<) B) Aged Patients (65>) C) HR + 95% CI overlap

Supplementary Material 7: RR +/- 95% CI comparison across recurrent and newly diagnosed GBM analysis.

Supplementary Material 8: RR +/- 95% CI comparison across non-vaccine and vaccine based immunotherapies.

## Data Availability

All collected data are available upon reasonable written request to the corresponding author.
