# Supplementary material for: Glioblastoma immunotherapy in the context of the aging immune system: a systematic review and meta-analysis
Source: J Neurooncol. 2026 Jan 12;176(2):164. doi: 10.1007/s11060-025-05395-1 (PMC12795865; doi:10.1007/s11060-025-05395-1)
Supplement: Supplementary file 1 — Supplementary Material 1: Inclusion/exclusion criteria for the meta-analysis and search engine terms. [file 11060_2025_5395_MOESM1_ESM.docx]

*Aged immunotherapy meta-analysis new search criteria*

### Inclusion

- All clinical trials of GBM utilizing immunotherapy with or without chemotherapy and radiation between Jan 1^st^ 2000 and April 1^st^ 2025.
- Published Phase I, II, III and IV, trials designated as such explicitly in the text
- Immunotherapies included: Oncolytic Virus, Tumor Vaccines (cells or peptides), Checkpoint inhibitors, CAR-T cells, Small Molecule or Antibody Inhibitors of Immune Pathways.
- Trials must include either individualized survival data separated by age for overall survival.
- Trials in setting of both primary and/or recurrent GBM with no other neurological diseases present.
- Ages 18+ only.
- Published in English (regardless of location conducted).
- Trials in Humans only.

### Exclusion

- Individual age data for all participants not available in manuscript or supplement.
- Tumor other than primary or recurrent GBM not delineated in individualized data.
- Presence of other neurological diseases in the study population.
- Published in language other than English.
- Trial not limited to human subjects.
- Participants aged less than 18.
- Trial is not published or not explicitly defined as Phase I, II, III, IV.

### Search Terms

Use Search Terms across Pubmed, Cochrane, (filter for clinical trials phase, II, III or IV and date range) (do not select randomized controlled trial filter)

Pubmed terms:

· glioblastoma and immunotherapy:

· glioblastoma and virus:

· glioblastoma and cellular vaccination:

· glioblastoma and peptide vaccination:

· glioblastoma and checkpoint inhibitors:

· glioblastoma and PD-1:

· glioblastoma and CTLA-4:

· glioblastoma and cytokine therapy:

· glioblastoma and CAR-T:

· glioblastoma and immune stimulation:

· glioblastoma and immune microenvironment:

· glioblastoma and dendritic cell vaccination:

·
