# Supplementary material for: Glioblastoma immunotherapy in the context of the aging immune system: a systematic review and meta-analysis
Source: J Neurooncol. 2026 Jan 12;176(2):164. doi: 10.1007/s11060-025-05395-1 (PMC12795865; doi:10.1007/s11060-025-05395-1)
Supplement: Supplementary file 2 — Supplementary Material 2: Phase III lung cancer immnunotherapy trials examined for age bias [file 11060_2025_5395_MOESM2_ESM.docx]

| **Author (first)** | **Year** | **% of aged patients in trial** | **mean/median age** | **total N** |
| --- | --- | --- | --- | --- |
| Gandara | 2021 | 46 | NA | 823 |
| Reck | 2021 | 51 | 65 | 361 |
| Reck | 2021 | 50 | 65 | 358 |
| Spigel | 2021 | 35 | 62 | 284 |
| Spigel | 2021 | 37 | 61 | 285 |
| Lee | 2023 | 74 | 75 | 302 |
| Lee | 2023 | 71 | 75 | 151 |
| Peters | 2022 | 57 | 66 | 234 |
| Peters | 2022 | 57 | 66 | 237 |
| Peters | 2022 | 50 | 65 | 145 |
| Peters | 2022 | 55 | 66 | 146 |
| Paz-Ares | 2025 | 51 | 65 | 242 |
|  | 2025 | 63 | 67 | 241 |
|  |  |  |  |  |
