# Supplementary figures and images for: Glioblastoma immunotherapy in the context of the aging immune system: a systematic review and meta-analysis
Source: J Neurooncol. 2026 Jan 12;176(2):164. doi: 10.1007/s11060-025-05395-1 (PMC12795865; doi:10.1007/s11060-025-05395-1)

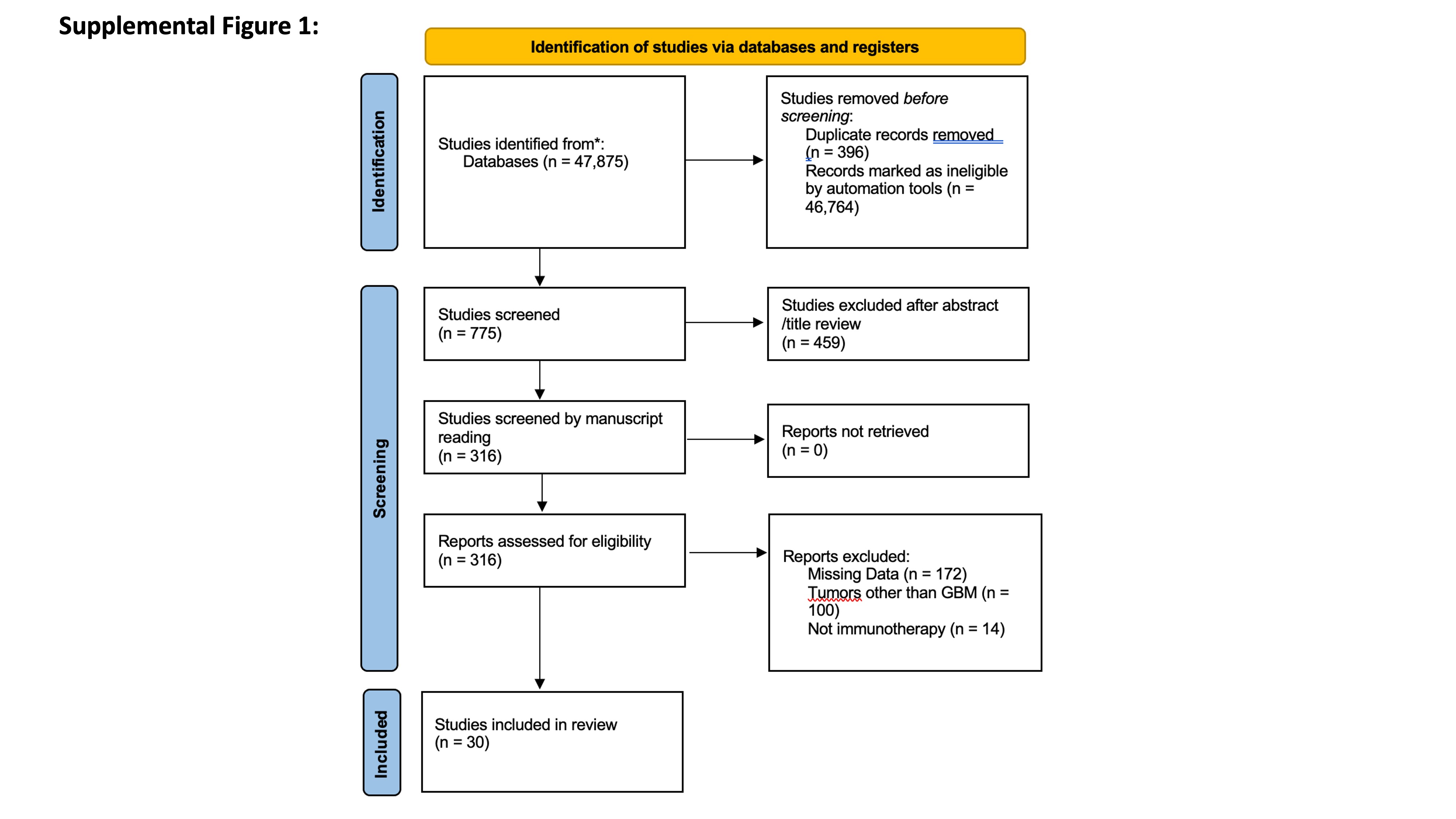

Supplement: Supplementary file 3 — Supplementary Material 3: PRISMA flow chart. [file 11060_2025_5395_MOESM3_ESM.jpeg]

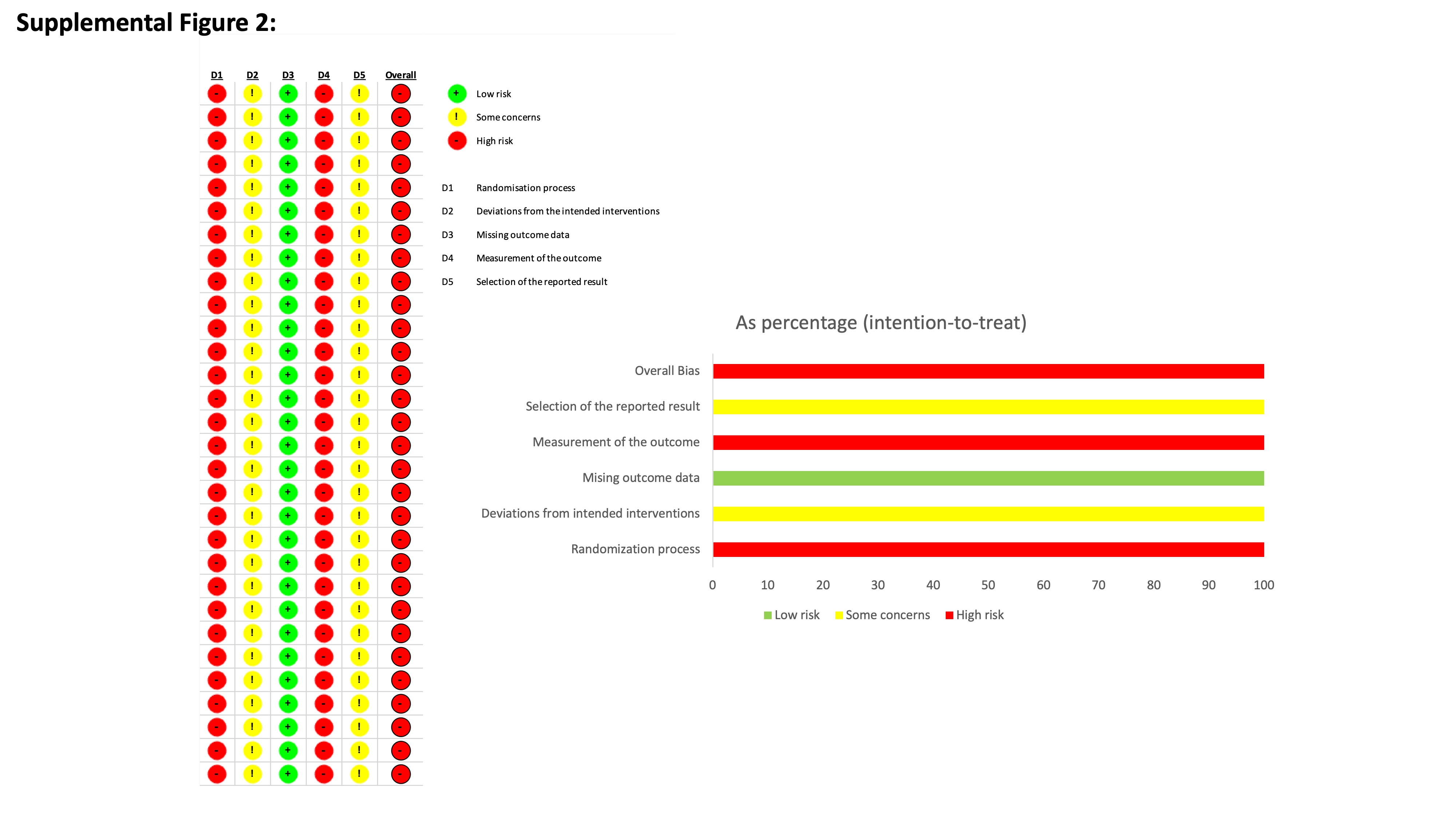

Supplement: Supplementary file 4 — Supplementary Material 4: Cochrane Risk of Bias Analysis. [file 11060_2025_5395_MOESM4_ESM.jpeg]

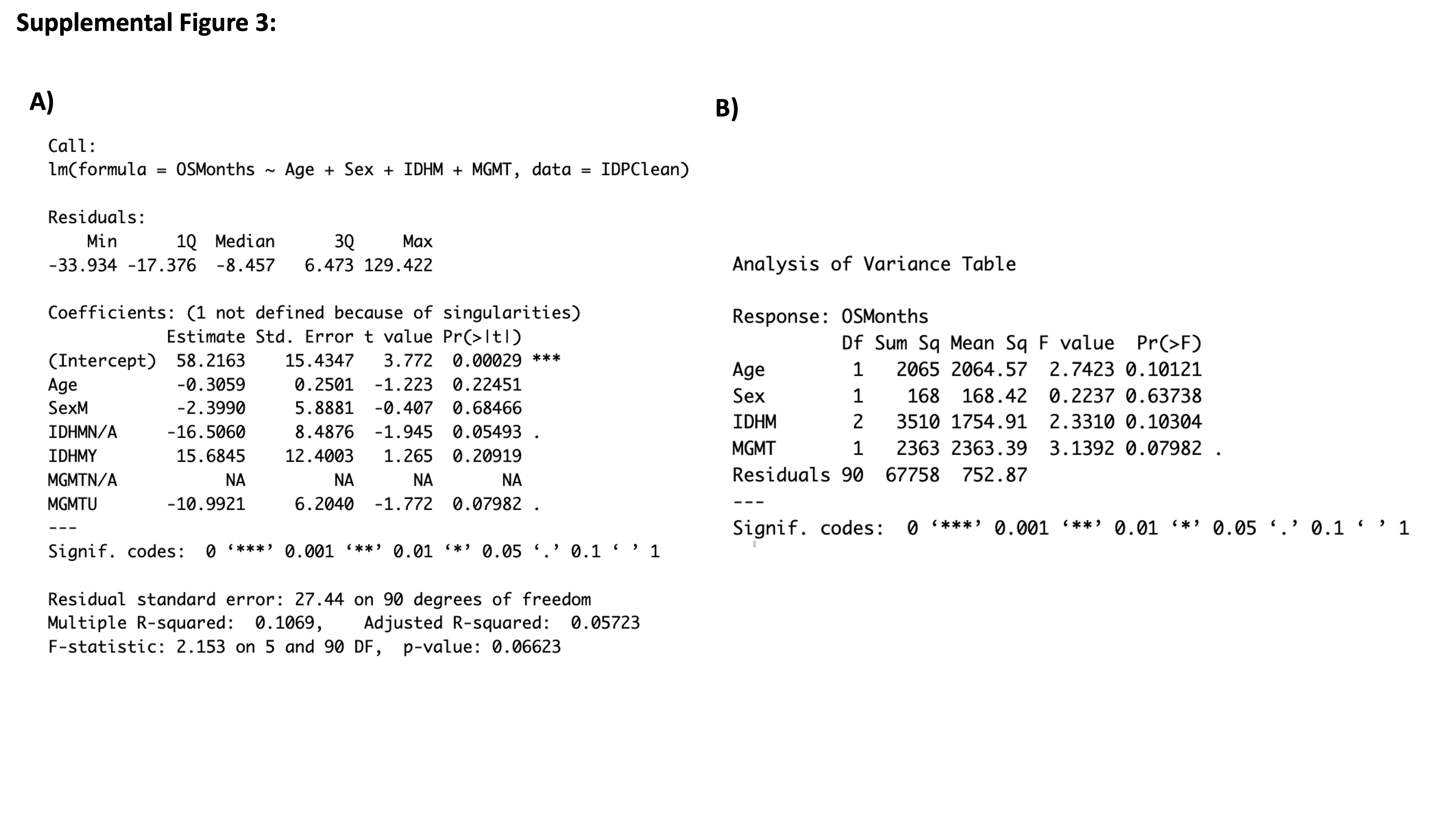

Supplement: Supplementary file 5 — Supplementary Material 5: Linear regression analysis of predictors for OS in GBM immunotherapy trials. A) Regression Analysis B) ANOVA [file 11060_2025_5395_MOESM5_ESM.jpeg]

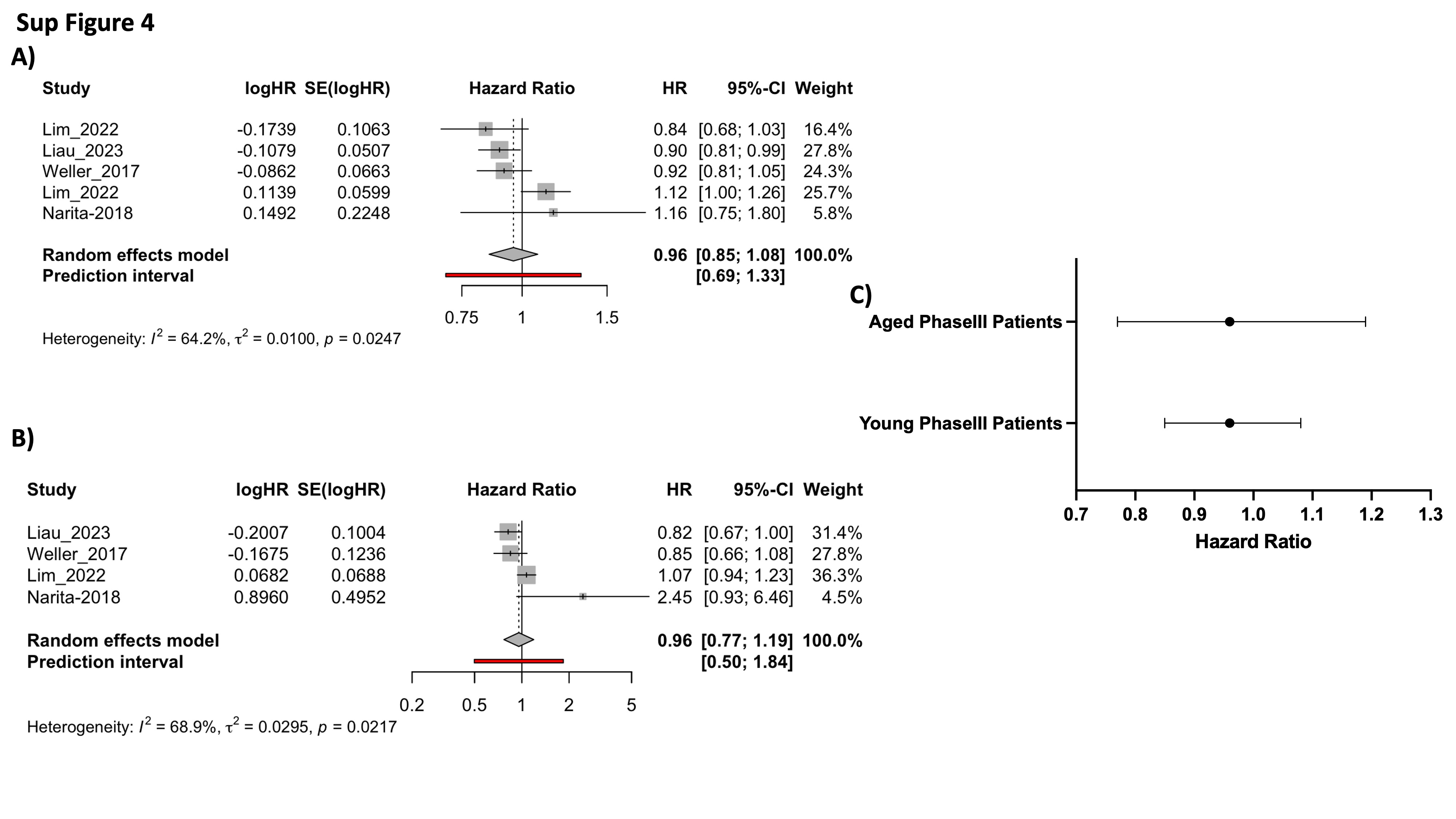

Supplement: Supplementary file 6 — Supplementary Material 6: HR comparison of Phase III clinical trials including an age bin analysis. A) Young Patients (65<) B) Aged Patients (65>) C) HR + 95% CI overlap [file 11060_2025_5395_MOESM6_ESM.jpeg]

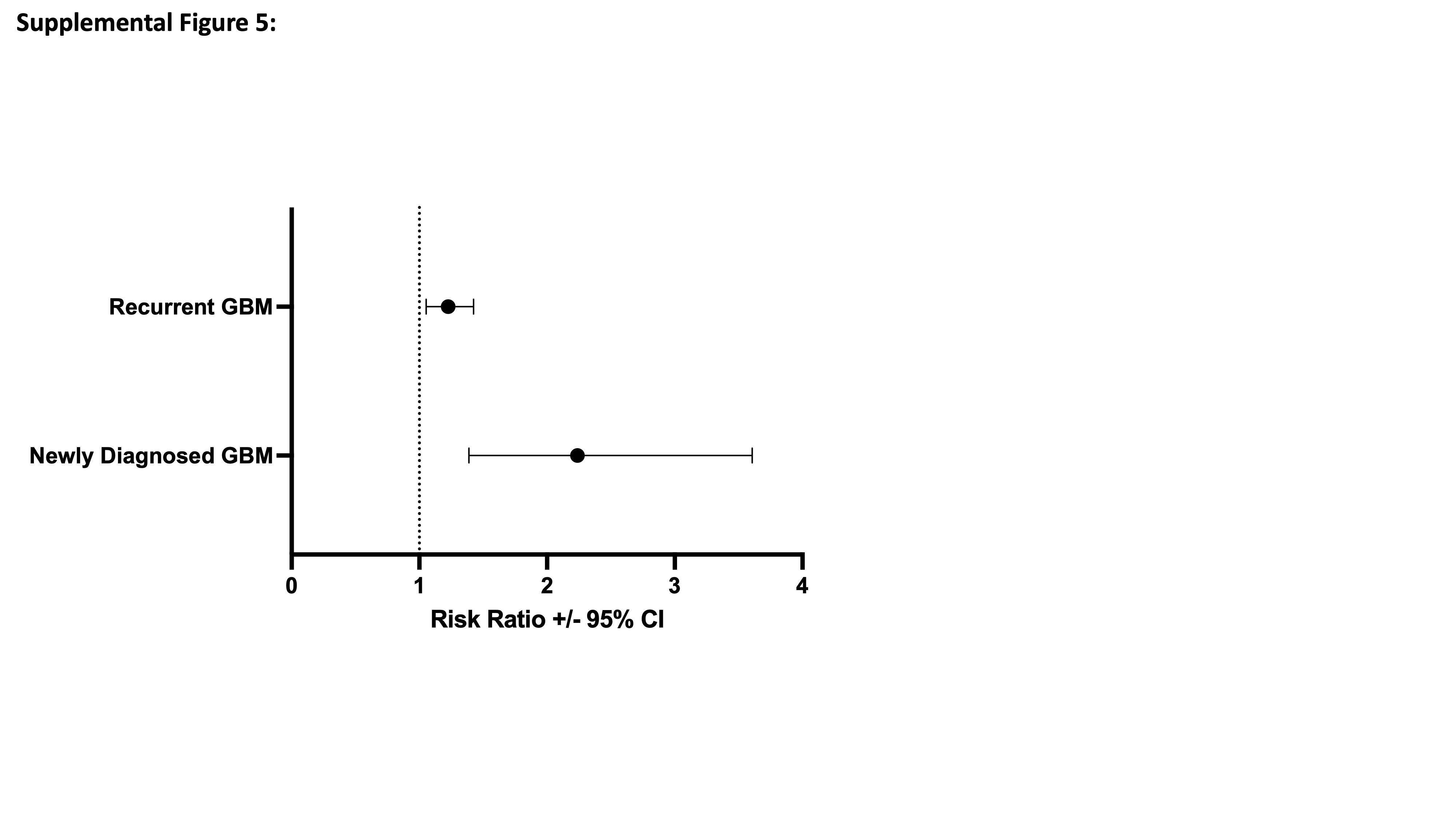

Supplement: Supplementary file 7 — Supplementary Material 7: RR +/- 95% CI comparison across recurrent and newly diagnosed GBM analysis. [file 11060_2025_5395_MOESM7_ESM.jpeg]

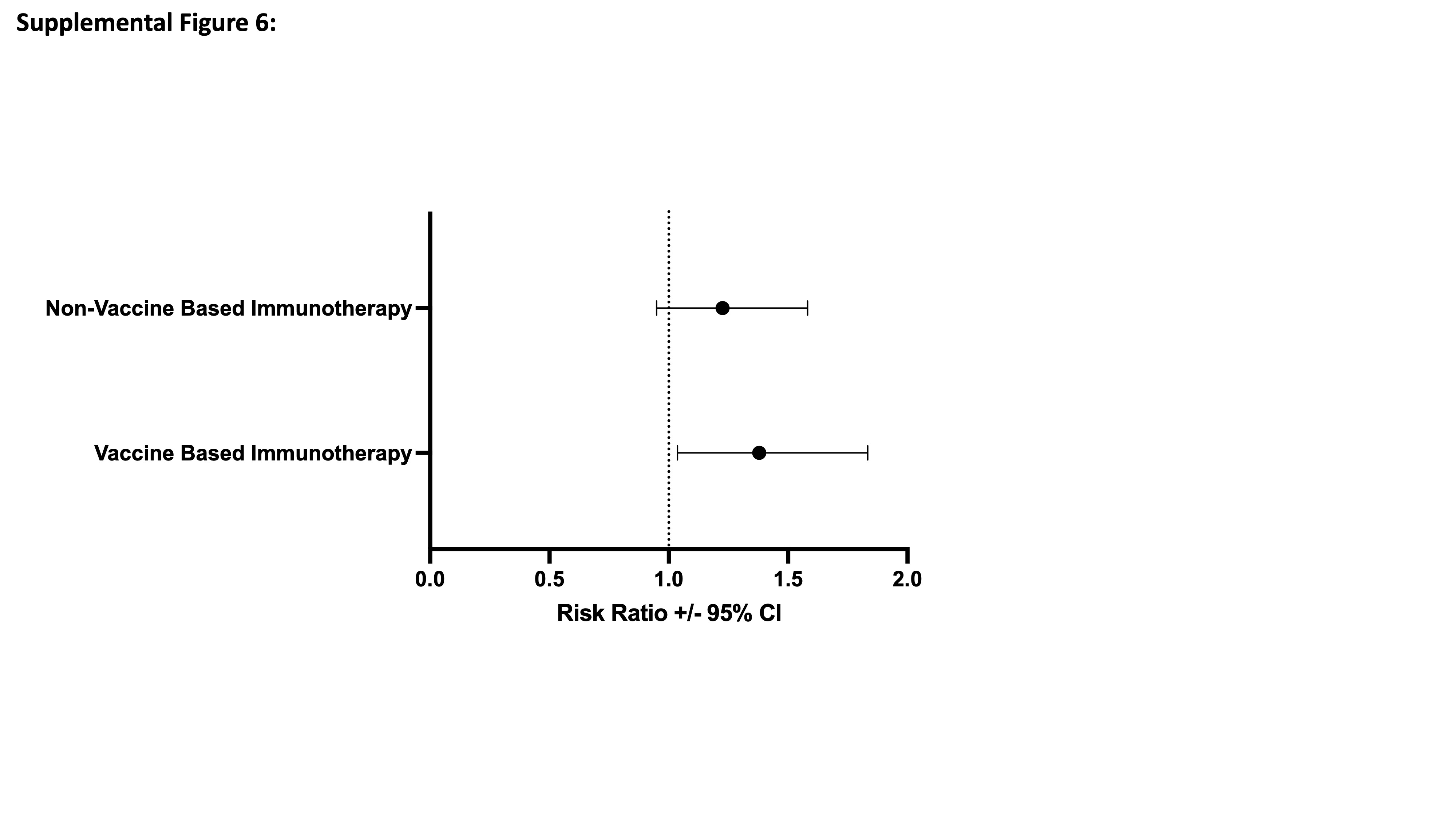

Supplement: Supplementary file 8 — Supplementary Material 8: RR +/- 95% CI comparison across non-vaccine and vaccine based immunotherapies. [file 11060_2025_5395_MOESM8_ESM.jpeg]
